# Bioinformatics-Based Identification of MicroRNA-Regulated and Rheumatoid Arthritis-Associated Genes

**DOI:** 10.1371/journal.pone.0137551

**Published:** 2015-09-11

**Authors:** Yi-Jiang Song, Guiling Li, Jian-Hua He, Yao Guo, Li Yang

**Affiliations:** 1 Key Laboratory of Biorheological Science and Technology, Ministry of Education, Bioengineering College, Chongqing University, Chongqing, China; 2 Institute of Genomic Medicine, Wenzhou Medical University, Wenzhou, Zhejiang Province, China; University of California, Los Angeles, UNITED STATES

## Abstract

MicroRNAs (miRNAs) act as epigenetic markers and regulate the expression of their target genes, including those characterized as regulators in autoimmune diseases. Rheumatoid arthritis (RA) is one of the most common autoimmune diseases. The potential roles of miRNA-regulated genes in RA pathogenesis have greatly aroused the interest of clinicians and researchers in recent years. In the current study, RA-related miRNAs records were obtained from PubMed through conditional literature retrieval. After analyzing the selected records, miRNA targeted genes were predicted. We identified 14 RA-associated miRNAs, and their sub-analysis in 5 microarray or RNA sequencing (RNA-seq) datasets was performed. The microarray and RNA-seq data of RA were also downloaded from NCBI Gene Expression Omnibus (GEO) and Sequence Read Archive (SRA), analyzed, and annotated. Using a bioinformatics approach, we identified a series of differentially expressed genes (DEGs) by comparing studies on RA and the controls. The RA-related gene expression profile was thus obtained and the expression of miRNA-regulated genes was analyzed. After functional annotation analysis, we found GO molecular function (MF) terms significantly enriched in calcium ion binding (GO: 0005509). Moreover, some novel dysregulated target genes were identified in RA through integrated analysis of miRNA/mRNA expression. The result revealed that the expression of a number of genes, including *ROR2*, *ABI3BP*, *SMOC2*, etc., was not only affected by dysregulated miRNAs, but also altered in RA. Our findings indicate that there is a close association between negatively correlated mRNA/miRNA pairs and RA. These findings may be applied to identify genetic markers for RA diagnosis and treatment in the future.

## Introduction

Rheumatoid arthritis (RA) is a highly prevalent chronic immune-mediated inflammatory disease, principally leading to injury of the synovial joint, as well as affecting many other tissues [1, 2]. In the developed world approximately 0.5%-1% of adults suffer from joint and other bone pains during their lifetime, leading to chronic disability or even death. RA is characterized by systemic inflammation and persistent synovitis, and it often leads to other serious complications as well. Several groups have reported increased risk of fatal myocardial infarction, lung cancer, and other extra-articular complications in RA patients [2–5]. Synovial fibroblasts (SFs), also known as fibroblast-like synoviocytes, are a special cell type located inside joints in the synovium, and they play a crucial role in the pathogenesis of RA [6, 7]. RA is also characterized by increased levels of SF-secreted proinflammatory cytokines that promote extracellular matrix degradation, chemokine production, and progressive destruction of joint membrane, cartilage and bone [8]. Moreover, RA-associated SFs (RASFs) secrete growth factors to avoid apoptotic cell death, and they promote angiogenesis [9]. However, in contrast to other arthropathies such as osteoarthritis (OA), the mechanisms that trigger the aggressive behavior of SFs in RA have not been elucidated clearly. OA is a degenerative disorder that shows multiple clinical manifestations or symptoms resembling those of RA. Therefore, OA patients are often chosen as controls in RA studies [10].

Development of new therapies using genetic or biological approaches enables better diagnosis and treatment of chronic diseases. Recent years have witnessed unprecedented attention on the role of epigenetic regulation in the pathogenesis of autoimmune disease [11, 12]. Epigenetic studies mainly focus on DNA methylation, microRNA (miRNA) regulation, and histone modifications of gene expression profiles and their effects on complicated biological processes, including development of chronic inflammation [13]. Genome-wide analyses of the epigenome have enabled identification of novel genes involved in RA pathogenesis, and the identified epigenetic biomarkers may be important in the context of a therapeutic regimen that targets active RASFs [14, 15]. Compared to normal OASFs, RASFs exhibit global genomic hypomethylation, which in turn affects the expression of proinflammatory cytokines and growth factors [16, 17]. Global DNA hypomethylation has also been observed in T cells and peripheral blood mononuclear cells (PBMCs) from RA patients [18, 19]. The hypomethylating milieu may induce irreversible phenotypic changes in normal SFs. Recently, it has been reported that miRNAs are involved in the pathogenesis of RA, and that they are important for enduring the activation and aggressiveness of SFs [20]. Recent advances have also indicated that miRNAs play a critical role in the pathogenesis of inflammatory diseases including RA [21, 22]. However, as a novel therapeutic target, the biological significance of miRNAs in RA pathogenesis has been underestimated.

miRNAs are a class of small non-coding RNAs (approximately 22–23 nucleotides in length) that play important roles in post-transcriptional gene regulation. In eukaryotic cells, miRNAs bind to the 3′-untranslated regions of the target mRNAs, resulting in translational repression and gene silencing [23, 24]. Changes in the miRNA expression profile may lead to gene dysregulation, and even associated phenotypic aberrance. miRNAs have been considered potential biomarkers for RA [25, 26]. Tumor necrosis factor (TNF)-α is one of the major proinflammatory cytokines involved in the pathogenesis of RA[27]. Trenkmann et al. demonstrated that miR-18a plays an important role in the TNF-α-mediated signaling pathway and that it is a component of the positive regulatory feedback loop in the nuclear factor kappa-light-chain-enhancer of activated B cells (NF-κB) signaling pathway in RASFs [28]. Semaan and Frenzel showed that miR-346 controls TNF-α synthesis by regulating tristetraprolin expression in lipopolysaccharide-activated RASFs [29]. Moreover miR-155 suppresses the expression of *SOCS1*, which may lead to upregulation of *TNF-α* and interleukin (IL)-1β in RA PBMCs [30]. miRNA dysregulation has been detected in SFs, PBMCs, plasma, and T cells. miR-146a is significantly upregulated in RA synovial tissue, PBMCs, and CD4+ T cells, and the expression change is closely correlated with TNF-α level [31–33]. miR-24, miR-26a, and miR-125a-5p are present in high concentrations in the plasma of RA patients, compared with healthy control (HC) individuals, indicating that these miRNAs might be RA plasma biomarkers [34]. Zhu S et al. reported that miR-23b suppresses *IL-17*-associated autoimmune inflammation by targeting *TAB2*, *TAB3*, and *IKK-α* [35].

Driven by technological advances, recent years have witnessed the application of a series of new methods for different aspects of disease research. Along with the development of microarray and next-generation sequencing technologies (NGS), reduced cost and increased data throughput have enabled the application of high-throughput technologies in new areas of life science research [36, 37]. The advent of new technologies has enabled clinical application of microarray or NGS for the study of hereditary diseases. High throughput analytical methods have become widely applicable to human disease-related studies. RA is heterogeneous in nature and it is influenced by variations in environmental factors and polygenic background. This heterogeneity is one of the main reasons that RA treatment is difficult [38]. Wright et al. have successfully applied RNA-seq analysis of RA neutrophils to identify the pre-therapy IFN-regulated gene expression profile that correlates with optimal response to TNF-inhibitor therapy [39].

To minimize heterogeneity and to overcome the limitations of a single research project, we employed both literature review and data mining in the current study. Both microarray and RNA-seq data from RA-related studies were collected to identify miRNA-regulated differentially expressed genes (DEGs). We identified RA-associated miRNAs from literature, and compared their target gene expression profiles between RA and OA or HC samples. The identification of RA epigenetic biomarkers may allow better diagnosis and treatment of RA, and eventually, provide opportunities to personalize rheumatic disease management.

## Methods

### Data mining of RASFs-associated miRNAs and their conventional analysis

A PubMed advanced search was performed for publications up to June 2014, using “microRNA” and “rheumatoid arthritis” as key words in the Title/Abstract field, without restriction on language or article type. Study organisms other than human patients were excluded. RASFs-relevant studies were identified using the search words by “synoviocyte”, “fibroblast-like synoviocyte”, or “synovial fibroblast”. The research project was designed to enable comparison between RA and HC (or OA) patients.

When searched by name or key word, information including family, genomic coordinates, clustering, references, etc., can be obtained from miRBase. Although currently there are many miRNA target gene prediction software, their algorithms may not be the same and each has its own advantages and disadvantages. Therefore, different software are frequently used in combination to reduce errors. In the current study, miRNA target genes were predicted using TargetScan v 6.2 and miRDB v 5.0 with different algorithms. TargetScan (http://www.targetscan.org/) predicts the regulatory targets of vertebrate miRNAs by searching for conserved 7-8mer sites that match the seed region of each miRNA [40]. miRDB (http://www.mirdb.org/miRDB/) predicts miRNA target genes based on support vector machines and public high-throughput experimental data [41]. To reduce false-positive results, only genes pulled out by both methods were selected as miRNA targets for subsequent analysis. miRDB v 5.0 utilizes miRNA data provided by miRBase v 21, while TargetScan v 6.2 utilizes an older version of miRBase v 17. Therefore, if a particular miRNA prediction data was missing in TargetScan, we used the prediction results from miRDB.

### Data collection of RA-associated genes

NCBI Gene Expression Omnibus (GEO) datasets were searched using “Rheumatoid Arthritis” as the key word and it returned 1,952 entries (updated up to June 2014), among which 113 were data series. A GEO series links a set of related samples together, providing a description, summarized conclusions, and the original raw data files of the overall study. These 113 data series were used as the original datasets, and they were subjected to a series of follow-up screening to identify the final datasets.

GEO provides a large collection of microarray expression data, while Sequence Read Archive (SRA) provides substantial sequencing data. Belonging to NCBI, SRA collects raw sequencing data from next-generation sequencing platforms, and all submitted databases can be accessed for free. We searched SRA using the key words “Rheumatoid Arthritis” and we found only three valid records. The human gut metagenome record was excluded. The remaining two were from the miR-seq and RNA-seq datasets. The SRA data were converted to FASTQ format raw sequencing data using the SRA Toolkit v 2.3.5, and then further analyzed and compared with the data obtained from GEO.

### Analysis of GEO microarray data

For consistency, all microarray datasets were analyzed individually using the same analytical tool, GEO2R. GEO2R (http://www.ncbi.nlm.nih.gov/geo/geo2r/) is an R-based interactive web tool to identify genes that are differently expressed under various experimental conditions. GEO2R uses metadata columns to categorize defined sample groups. Not all samples may be required for each analysis. Therefore, before assigning sample groups, each sample introduction was reviewed before the samples were selected for follow-up analysis. Altered gene expressions exhibiting changes of at least 2-fold and with *p*-values of < 0.05 were considered statistically significant.

### Analysis of SRA sequencing data

In order to obtain high quality sequencing data, the RNA sequences were first subjected to data cleaning by eliminating low quality reads and various read contaminants such as adapter sequences. A quality control (QC) toolkit, NGS QC Tools (http://www.nipgr.res.in/ngsqctoolkit.html) v 2.3, was applied for the NGS data [42]. Then, Bowtie-integrated TopHat (http://ccb.jhu.edu/software/tophat/index.shtml) v 2.0.6 was used to map RNA sequences from the spliced transcripts to the UCSC human genome (hg19) [43]. Upon mapping RNA-seq reads to the human genome, TopHat identified potential exons and built a database of possible splice junctions.

Following genomic alignment, the Cufflinks package v 2.1.1 was used for transcript assembly, and analyses of differential expression and regulation (http://cole-trapnell-lab.github.io/cufflinks/) [44]. Each sample assembly file was merged together with the reference annotation file downloaded from Ensembl using Cuffmerge and included in the final output. The other sub-program, Cuffdiff, was then used to calculate differences in expression at the gene and transcript levels, as well as alternative promoter usage and splicing. Differential gene expression was calculated as the ratio of the RA group to the control group for every gene or transcript with statistically significant values [45].

### Functional annotation of significantly dysregulated genes

The dysregulated genes between RA and control samples were extracted as DEGs. These genes needed functional annotation. Only genes that exhibited significant expression differences (*p*-value < 0.05 and fold change > 2) were functionally annotated. The genes were analyzed using Database for Annotation, Visualization, and Integrated Discovery (DAVID) v 6.7, which supports online functional annotation, gene ID conversion, and other tools (http://david.abcc.ncifcrf.gov/) [46]. This functional annotation tool searched for functional enrichment clustering, pathway mapping, gene-disease association, etc. The list of official gene symbols was submitted via the upload interface, and the species was specified as *Homo sapiens*. Kyoto Encyclopedia of Genes and Genomes (KEGG) pathway and Gene Ontology (GO) were chosen as the functional annotation categories for this analysis [47]. Cytoscape (http://www.cytoscape.org/) was applied to identify miRNA/mRNA co-expression interaction networks [48].

## Results

### Microarray and raw sequencing data collection

**Fig 1:**
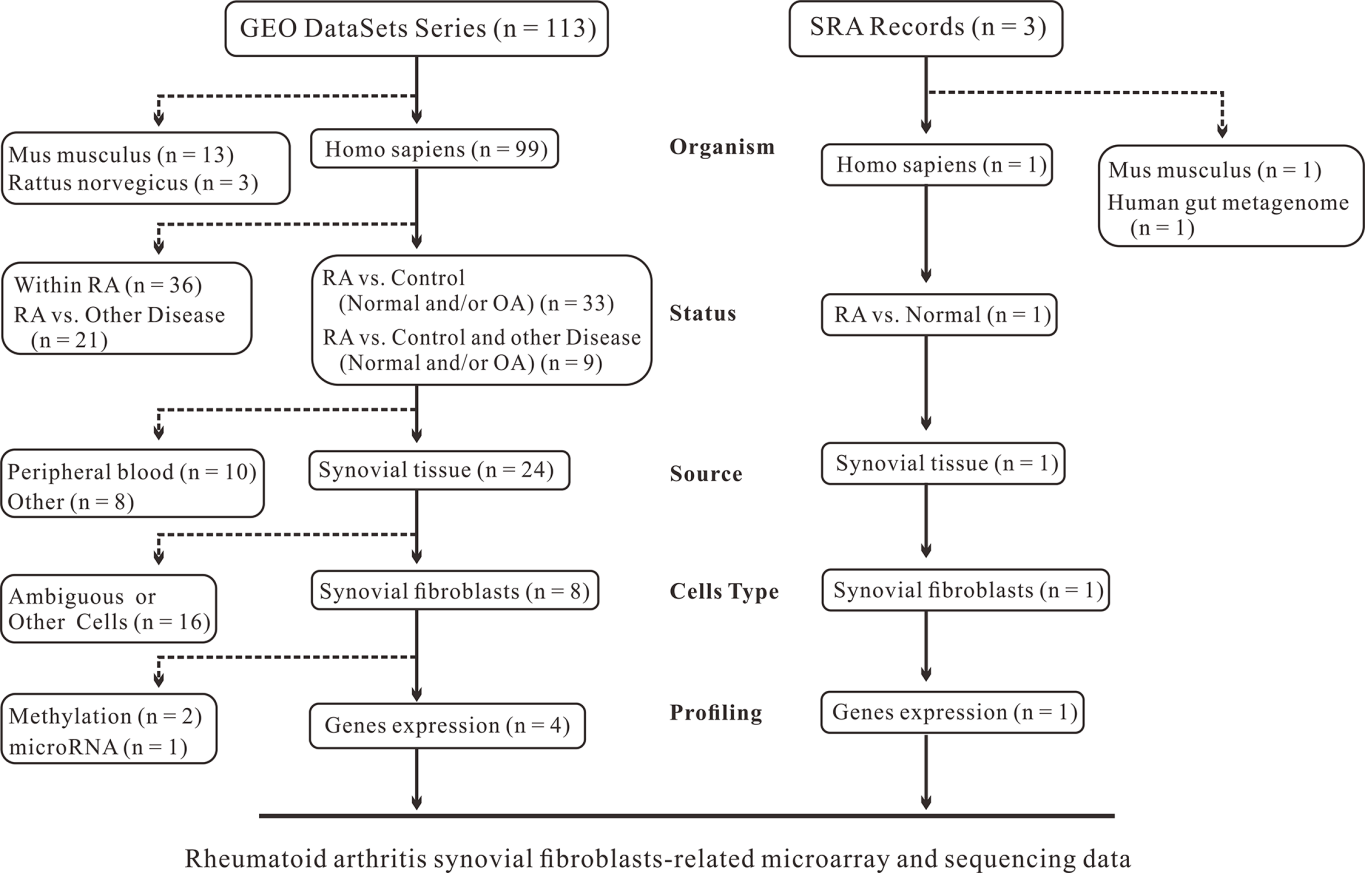
Flow chart depicting the study selection protocol for rheumatoid arthritis (RA) gene expression.

We filtered the data using various parameters, including organism, experimental object, and cell source, and collected a series of GEO microarray data, as well as raw sequencing data of RA in comparison with HC (or OA). A flow chart depicting the detailed study selection protocol is shown in Fig 1. We selected 4 microarray series (GSE7669 [49], GSE49604 [50], GSE21959 [51], and GSE29746 [52]) from the literature. However, not all the samples in each series were subjected to subsequent analysis. For example, only samples without drug treatment were reserved in GSE49604, and all hypoxia-treated samples were abandoned in GSE21959. Each series contained one dataset, except for GSE29746, which had three parts of data from RA, OA, and HC and thus, consisted of two datasets (RA vs. OA, RA vs. HC). In addition to GEO microarray data, RNA-seq data SRP009315 [53] from SRA was obtained. Thus, datasets from both microarray and sequencing types were collected.

### RASF gene expression profiling and functional annotation by microarray

The microarray data was divided into two different comparison groups, RA vs. HC, and RA vs. OA. R-based GEO2R was used to analyze the GEO microarray data. For each series of data, DEGs were extracted and functionally annotated. The corresponding biological processes that these genes are involved in were identified by functional enrichment analysis using DAVID v 6.7. The results for each dataset are outlined below.

Series GSE29746: The RA datasets were first compared with the HC datasets. One hundred and forty-two genes showed at least 2-fold expression change (*p* < 0.05). While 3,880 genes showed differential expression between RA and OA. Among them, 123 genes show different expression in both comparison groups. The corresponding DEGs of each group were then subjected to GO analysis separately, and were divided into several major categories (S1 Table). The highly enriched Molecular Function (MF) terms, including calcium ion binding (GO: 0005509), channel activity (GO: 0015267), passive transmembrane transporter activity (GO: 0022803), substrate specific channel activity (GO: 0022838), ion channel activity (GO: 0005216) etc., were identified for both groups. To further characterize these biological pathways, the genes were also subjected to KEGG pathway analysis. The results revealed that two pathways, hsa04514: cell adhesion molecules (CAMs) and hsa00982: drug metabolism, were significantly enriched in both the RA/OA and RA/HC datasets. OA and HC were both used as the control for RA, however, gene expression may differ between OA and HC. The potential RA-related genes identified from both groups were further examined for the expression between OA and HC. And those exhibit different expression in OA/HC dataset were considered false positives and removed from the candidate list. Consequently, 117 and 1882 DEGs remained in RA/HC and RA/OA comparison groups, respectively. In addition, *ARHGAP15*, *FMO3*, *LRRC4B*, *ROR2*, and a number of other genes were differentially expressed in both datasets. Functional annotation of these DEGs showed that they were highly enriched in calcium ion binding, carbohydrate binding, gated channel activity, metal ion transmembrane transporter activity, while the false positives are not involved in these pathways. However, the false positives and the DEGs are both enriched in cation channel activity, sugar binding, gated channel activity, carbohydrate binding and metal ion transmembrane transporter activity, probably due to the high similarities in the genetic background and clinical symptoms between RA and OA.

Series GSE21959: Hypoxic-treated samples were eliminated before analysis. Eight hundred and nineteen genes were found to be differentially expressed between the RA and HC groups. Their highly enriched GO MF terms included calcium ion binding (GO: 0005509, *p*-value = 0.001), protein dimerization activity (GO: 0046983, *p*-value = 0), and identical protein binding (GO: 0042802, *p*-value = 0.052). Some highly enriched KEGG pathways were also detected (S2 Table), including pathways in cancer (hsa05200, *p*-value = 0.051), cytokine-cytokine receptor interaction (hsa04060, *p*-value = 0.032), neuroactive ligand-receptor interaction (hsa04080, *p*-value = 0.046), and calcium signaling pathway (hsa04020, *p*-value = 0.026). Because series GSE29746 and GSE21959 shared the same platform (GPL4133), and contained both RA and HC datasets, they were cross-compared. The results revealed that *ADAM2*, *DYNAP*, *ICAM2*, *IFI27*, *IL27RA*, *NKX2-3*, and a few other genes were differentially expressed in both series with the same variation trend.

Series GSE7669: We identified a total of 320 DEGs that exhibited at least 2-fold (*p* < 0.05) expression change between the RA and OA groups. A series of GO terms were then identified (S3 Table), and the most enriched GO MF term was ion binding (GO: 0043167, *p*-value = 0.061). The genes were KEGG-annotated and categorized as focal adhesion (hsa04510, *p*-value = 0.002), neuroactive ligand-receptor interaction (hsa04080, *p*-value = 0.072), mitogen-activated protein kinases (MAPK) signaling pathway (hsa04010, *p*-value = 0.090), etc. We further set the threshold of false discovery rate (FDR)-adjusted *p*-value at 0.05 and specified at least 2-fold change, and found that *PRG4* was significantly downregulated in RA, compared with OA.

Series GSE49604: This series of data is derived from two platforms (GPL8432 and GPL10558), which represent the global gene expression profiles of synoviocytes and macrophages of RA and OA, after eliminating the data for synovial fluid macrophages and PBMCs in platform GPL8432. Moreover, to unify all the datasets, 6 drug-treated samples from platform GPL10558 were not taken into account. Thirty-six DEGs were identified from this series. Among them, *MBP* and *IFI6* were significantly differentially expressed (FDR-adjusted *p*-value < 0.05 and fold-change > 2) in RA. Because the RA/OA data comparison was performed for both GSE7669 and GSE49604 series, a merge analysis was performed with both series. It turned out that *CNN1*, *POSTN*, *RARRES2*, *MX1*, *THBS4*, and *TSPAN7* were differentially expressed in the RA group of both series.

### RASF gene expression profiling and functional annotation by RNA-seq analysis

Four sample data (2 RA and 2 HC) were extracted from SRP009315, and each data had 40,391,131–44,878,863 raw clusters. NGSQC Toolkits were used to clean the raw sequencing data. The cut-off value for Phred quality score was set at 20 and the cut-off percentage of high quality reads was 80%. Contaminant reads were discarded to get final clean reads. All the high quality reads were subjected to follow-up analysis. Alignment of the sequencing reads to a reference genome is a core step in RNA-seq analysis workflows. The reads were aligned using TopHat, and the results revealed that 96% of the reads in each sample matched those in the human genome. After analyzing TopHat log files, less than 0.1% of the total reads were removed from the genome mapping data because of low quality.

The Cufflinks package consists of a number of tools for RNA-seq dataset analysis. Cufflinks was used to assemble the transcripts and assess their abundance. After mapping the sequencing reads to the reference genome with TopHat, the transcripts were assembled and their relative expression levels were calculated using Cufflinks and represented in terms of fragments per kilobase of exon per million fragments mapped (FPKM) [54]. Cuffmerge was used to merge several assemblies into one file. Cuffdiff was used to test differential expression and regulation in the RNA-seq samples. The expression of 138 genes was significantly altered in RASFs, with at least 2-fold change and FDR-adjusted *p*-values of < 0.05. GO and KEGG pathway analyses were performed using DAVID to analyze the genes at the functional level (S4 Table). GO MF analysis revealed that the DEGs between RA and HC were significantly enriched in calcium ion binding (GO: 0005509, *p*-value = 0.026), carbohydrate binding (GO: 0030246, *p*-value = 0) and structural molecule activity (GO: 0005198, *p*-value = 0.004), etc. Additionally, significantly enriched KEGG pathways were mainly involved in hematopoietic cell lineage (hsa04640, *p*-value = 0.007), renin-angiotensin system (hsa04614, *p*-value = 0.010), tryptophan metabolism (hsa00380, *p*-value = 0.048) etc.

GSE21959 was selected as the representative microarray data. This series contained two groups of RA and HC samples. These data were then cross-compared with the sequencing data. Our results showed that the GO MF terms of both microarray and sequencing data were enriched in calcium ion binding (GO: 0005509) and carbohydrate binding (GO: 0030246). For biological processes (BP), the enriched GO terms corresponded to immune response (GO: 0006955) and cell adhesion (GO: 0007155). For cellular component (CC), the enriched GO terms corresponded to extracellular region (GO: 0005576). A large number of genes, including *ANK3*, *CMKLR1*, *F2RL2*, *IFI27*, *NES*, *OLFM2*, etc. were differentially expressed in both the datasets consistently.

### Literature screening for RASF-associated miRNAs and their target gene prediction

We performed a literature screening of 77 RA-associated miRNA studies, of which 20 were review articles. In the literature, aberrant expression of 14 miRNAs has been detected in RA samples. The expression of miR-146a, miR-155, miR-203a, miR-214, miR-221/222, miR-223, and miR-323 was higher in RASFs than that in OASFs or fibroblasts from HC. However, there is also evidence indicating decreased expression of miRNA miR-19b, miR-22, miR-23b, miR-30a, miR-34a* and miR-124a in RASFs (Table 1). Fisher's exact test was also performed to test the statistical significance of the common targets predicted by TargetScan and miRDB.

**Table 1:** Rheumatoid arthritis synovial fibroblast (RASF)-associated miRNAs and their predicted target gene numbers.

miRNA	Previous IDs	Expression^1^	TargetScan^2^	miRDB^3^	Common^4^	*P*.Value
**miR-19b-3p**	miR-19b	down↓^[^ ^57^ ^]^	1171	786	577	0.00
**miR-22-3p**	miR-22	down↓^[^ ^58^ ^]^	508	430	231	0.01
**miR-23b-3p**	miR-23b	down↓^[^ ^35^ ^]^	1127	866	527	0.00
**miR-30a-5p**	miR-30a	down↓^[^ ^35^ ^,^ ^59^ ^]^	1357	842	629	0.00
**miR-34a-3p**	miR-34a*	down↓^[^ ^60^ ^]^	-	233	233	-
**miR-124-3p**	miR-124a; miR-124	down↓^[^ ^61^ ^]^	1654	901	692	0.00
**miR-146a-5p**	miR-146; miR-146a	up↑^[^ ^31^ ^,^ ^62^ ^]^	224	224	60	1.00
**miR-155-5p**	miR-155	up↑^[^ ^63^ ^,^ ^62^ ^]^	440	311	143	0.00
**miR-203a-3p**	miR-203a	up↑^[^ ^63^ ^]^	868	711	313	0.00
**miR-214-3p**	miR-214	up↑^[^ ^35^ ^]^	678	790	251	0.04
**miR-221-3p**	miR-221	up↑^[^ ^56^ ^]^	446	316	161	0.00
**miR-222-3p**	miR-222	up↑^[^ ^56^ ^]^	446	320	159	0.00
**miR-223-3p**	miR-223	up↑^[^ ^64^ ^,^ ^65^ ^]^	311	242	99	0.03
**miR-323a-3p**	miR-323; miR-323-3p	up↑^[^ ^56^ ^]^	528	389	141	0.00

^1^ trend of expression change

^2^ numbers of predicted target genes in TargetScan

^3^ numbers of predicted target genes in miRDB

^4^ numbers of predicted target genes in both TargetScan and miRDB

miRNA annotation information, including miRNA family, genomic coordinates and cluster, were then retrieved from miRBase v 21. Expression analysis of different miRNA members within the same miRNA cluster or family might shed light on their functional relationship and the mechanism underlying their regulatory network [55]. Interestingly, miRNA-221 and miRNA-222 belong to not only the same cluster, but also the same family. A recent study of miRNA expression signatures in RA revealed that the level of miRNA-221/222 cluster significantly increases in SFs [56], indicating that the miRNA-221/222 cluster may be a disease progression marker in RA. Bioinformatics algorithms were then applied to predict miRNA target genes. To reduce the number of false-positives, only genes present in both the TargetScan and miRDB databases were selected. Fourteen miRNAs were differentially expressed in RASFs [35, 56–65], and their corresponding target genes were predicted (Table 1). However, to elucidate the miRNA/mRNA relationships in a pairwise manner, the expression profile of RA-associated genes needs to be analyzed (S1 Fig).

### Selection of negatively-correlated mRNA/miRNA pair candidates

**Fig 2:**
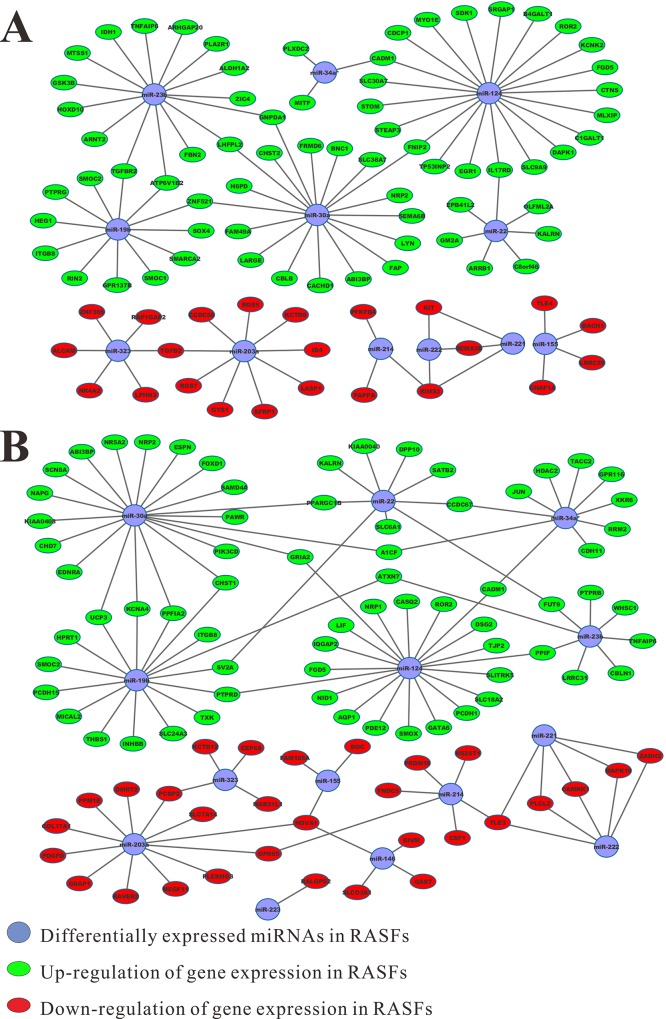
Genetic interaction networks for mRNA/microRNA (miRNA) pair candidates. The dysregulated RA-associated miRNAs are indicated in blue, and miRNAs targeting up and downregulated genes are represented by red and green, respectively. A) miRNA-mRNA interaction network from microarray data; B) miRNA-mRNA interaction network from RNA-seq data.

Recently several studies have reported the identification of RA-related miRNAs and their negatively-correlated target genes in RA. For instance, miR-23b was found downregulated in RA compared to HC, and its several target genes were upregulated in RA [35]. In addition, negative correlation of miR-19/TLR2, as well as miR-323 and its several target genes were found in RA [56]. In the current study, the potential target genes identified from microarray gene expression profile meet the following criteria: the genes show the same expression trend in all tested projects; the expression of miRNA and target genes are negatively correlated; and the target genes show differential expression (at least 2-fold change) in each comparison group. Thirteen putative target genes of dysregulated miRNAs were identified from the RA and HC datasets of the GSE21959 and GSE29746 series (Table 2). In addition to these criteria, target genes must show differential expression in at least one round of comparison. Using the above-mentioned criteria, 66 target genes were identified from the previous round of RA/HC comparison, while 39 putative miRNA target genes are identified from the RA and OA datasets of GSE29746, GSE49604, and GSE7669 (Table 3). Because the sample preparation, data collection, and analysis methods were different, the microarray and RNA-seq data were analyzed separately. Analysis of the RNA-seq data revealed that the expression of 91 genes was negatively correlated with dysregulated miRNAs. Therefore, these genes were considered putative miRNA target genes. Notably, variations in the expression of *ROR2*, *ABI3BP*, and *SMOC2* between the RA and HC datasets were consistent, as confirmed from both sequencing and microarray data. A few representative DEGs, sorted by their relative expression abundance, are listed in Table 4. After combining the gene expression profiles of the mRNA/miRNA pair candidates, their records were imported into Cytoscape for interaction analysis and visualization (Fig 2).

**Table 2:** Putative target genes identified from rheumatoid arthritis (RA)/healthy control (HC) microarray data.

Gene	miRNA	GSE21959		GSE29746	
		log FC	*P*.Value	log FC	*P*.Value
** ABI3BP**	miR-30a	1.394	0.012	1.304	0.054
** CCDC67**	miR-22; miR-34a*	1.102	0.052	1.398	0.016
** CHD7**	miR-30a	2.167	0.001	1.006	0.240
** DMRT2**	miR-203a	-4.259	0.000	-1.413	0.290
** DSG2**	miR-124	1.324	0.240	1.493	0.128
** ESPN**	miR-30a	1.676	0.049	1.118	0.207
** MAPK10**	miR-221; miR-222	-2.308	0.004	-1.488	0.116
** PAWR**	miR-30a	1.294	0.024	1.305	0.176
** PTPRB**	miR-23b	1.211	0.058	1.965	0.035
** ROR2**	miR-124	1.175	0.060	1.555	0.013
** SMOC2**	miR-19b	1.223	0.015	1.495	0.212
** UCP3**	miR-19b; miR-30a	1.266	0.154	1.041	0.243
** XKR6**	miR-34a*	1.378	0.057	1.092	0.277

**Table 3:** Putative target genes identified from rheumatoid arthritis (RA)/osteoarthritis (OA) microarray data.

Gene	miRNA	GSE29746		GSE7669		GSE49604	
		log FC	*P*.Value	log FC	*P*.Value	log FC	*P*.Value
**ATXN7**	miR-19b; miR-23b	1.479	0.026	0.183	0.697	0.189	0.226
**CADM1**	miR-124; miR-34a*	1.597	0.132	0.879	0.036	0.429	0.236
**CBLN1**	miR-23b	1.269	0.065	0.357	0.423	0.099	0.432
**CDH11**	miR-34a*	0.088	0.693	1.053	0.001	0.008	0.973
**CHST1**	miR-19b; miR-30a	1.216	0.213	0.108	0.608	0.077	0.521
**EDNRA**	miR-30a	0.584	0.170	1.468	0.036	0.249	0.272
**GATA6**	miR-124	0.258	0.642	1.086	0.098	0.008	0.952
**GPR116**	miR-34a*	1.743	0.001	0.036	0.909	0.006	0.962
**GRIA2**	miR-124; miR-30a	1.671	0.003	0.043	0.927	0.099	0.450
**HDAC2**	miR-34a*	1.079	0.001	0.347	0.177	0.145	0.207
**HPRT1**	miR-19b	1.173	0.020	0.090	0.641	0.172	0.251
**INHBB**	miR-19b	1.053	0.065	2.124	0.011	0.005	0.971
**JUN**	miR-34a*	1.246	0.002	0.692	0.169	0.235	0.170
**KCNA4**	miR-19b; miR-30a	1.242	0.390	0.426	0.507	0.028	0.847
**KIAA0040**	miR-22	1.104	0.112	0.546	0.247	0.175	0.135
**KIAA0408**	miR-30a	1.219	0.017	1.250	0.111	0.033	0.822
**LIF**	miR-124	1.201	0.031	0.366	0.708	0.046	0.752
**MICAL2**	miR-19b	1.975	0.000	1.216	0.001	0.005	0.987
**NAPG**	miR-30a	1.052	0.048	0.036	0.858	0.084	0.557
**NID1**	miR-124	0.448	0.540	1.038	0.005	0.044	0.726
**NR5A2**	miR-30a	2.038	0.009	0.135	0.845	0.144	0.227
**NRP1**	miR-124	1.061	0.051	0.863	0.040	0.164	0.232
**NRP2**	miR-30a	1.056	0.179	1.833	0.046	0.213	0.073
**PCDH1**	miR-124	1.885	0.001	0.906	0.040	0.054	0.632
**PDE12**	miR-124	0.456	0.385	1.047	0.044	0.103	0.383
**PIK3CD**	miR-30a	0.982	0.012	1.068	0.028	0.171	0.322
**PPFIA2**	miR-19b; miR-30a	1.785	0.000	0.191	0.428	0.166	0.204
**PPIF**	miR-124; miR-23b	1.339	0.013	0.145	0.633	0.110	0.322
**ROR2**	miR-124	2.545	0.000	0.679	0.064	0.351	0.270
**RRM2**	miR-34a*	0.757	0.227	1.214	0.005	0.591	0.019
**SAMD4A**	miR-30a	1.053	0.004	0.374	0.224	0.186	0.312
**SCN8A**	miR-30a	1.011	0.123	0.045	0.865	0.233	0.087
**SLC6A1**	miR-22	2.095	0.037	0.925	0.129	0.054	0.632
**SMOX**	miR-124	0.468	0.441	1.323	0.069	0.284	0.034
**SV2A**	miR-19b; miR-22	1.315	0.070	0.320	0.153	0.336	0.036
**TJP2**	miR-124	1.395	0.001	1.077	0.034	0.404	0.005
**TNFAIP6**	miR-23b	1.015	0.101	0.763	0.450	0.167	0.661
**TXK**	miR-19b	1.181	0.021	0.706	0.242	0.241	0.086
**WHSC1**	miR-23b	1.414	0.003	0.178	0.385	0.262	0.191

**Table 4:** Putative target genes identified from rheumatoid arthritis (RA)/healthy controls (HC) sequencing data.

Gene	miRNA	FPKM_HC	FPKM_RA	Log FC	*P*.Value
**LASP1**	miR-203a	1354.390	192.980	-2.811	0.037
**ALCAM**	miR-323	124.208	20.470	-2.601	0.002
**SFRP1**	miR-203a	97.100	4.420	-4.457	0.000
**LRRC59**	miR-155	91.001	24.123	-1.916	0.015
**GYS1**	miR-203a	76.312	19.463	-1.971	0.012
**B4GALT1**	miR-124	60.335	237.812	1.979	0.030
**KCTD9**	miR-203a	34.506	10.346	-1.738	0.019
**CCDC50**	miR-203a	33.295	9.508	-1.808	0.036
**ATP6V1B2**	miR-19b; miR-23b	32.409	120.477	1.894	0.016
**ID4**	miR-203a	28.005	3.079	-3.185	0.001
**STOM**	miR-124	27.766	152.788	2.460	0.003
**FAP**	miR-30a	14.053	105.083	2.903	0.001
**ABI3BP**	miR-30a	9.932	130.092	3.711	0.009
**SMOC2**	miR-19b	3.227	27.262	3.079	0.009
**ROR2**	miR-124	0.224	9.619	5.427	0.002

## Discussion

In this study, we gathered information on the differential expression of several genes between RA, OA, and HC. To overcome the high heterogeneity observed in the pathogenic mechanism of RA, data were procured from both microarray and RNA-seq datasets. To distinguish the DEGs in RA and HC (or OA), we examined differential expression profiles using microarray and RNA-seq analysis. We set the cut-off at > 2-fold change and the *p*-value at < 0.05. Among the analyzed DEGs, *COL4A5*, *IFI27*, *IFI6*, *NPTX1*, *PRG4*, *ROR2*, etc. were differentially expressed in RA-related gene expression profiles. To further analyze the biological roles of these DEGs, GO and KEGG analyses were performed using DAVID (S1–S4 Tables). Notably, calcium ion binding (GO: 0005509) was the most enriched GO MF term in almost all the datasets.

*COL4A5* encodes collagen IV, the major structural component of basement membranes, and we found that it is significantly downregulated (in all three microarray series: GSE7669, GSE21959, and GSE29746, with fold change > 2, p < 0.05) in RA. Petty RE, et al. reported the presence of antibodies against collagen IV in rheumatic diseases [66]. The interferon (IFN)-response gene *IFI27* exhibits significantly increased expression in patients a chronic autoimmune disease, Sjögren’s syndrome, compared with control individuals [67]. In our study, we found that *IFI27* was significantly upregulated (in both GSE21959 and GSE29746 series, with fold change > 2, p < 0.05) in the pair-wise comparisons between RA, OA, and HC. The IFN-inducible gene *IFI6* was also significantly dysregulated in RA PBMCs between nonresponders and responders to Tocilizumab [68]. These findings suggest that interferon signaling and apoptosis are involved in RA pathogenesis. *PRG4* encodes Lubricin, a boundary lubricant that is abundantly expressed in synovial fluid and articular cartilage. Lack of lubricin in the joint may lead to deficient joint lubrication and cartilage degradation [69]. Moreover, low levels of *PRG4* may be associated with strong synovial stromal activation [70].

In this study, we have identified a series of genes as putative molecular signatures of RA. These genes extend our understanding of RA disease mechanisms, and may have applications in potential biotherapeutic approaches for RA diagnosis and treatment.

A number of studies have reported the aberrant expression of miRNAs in RA, which indicates that miRNAs play important roles in RA. In the current study, we identified 14 miRNAs that might be important for RA pathogenesis and annotated their functions using information derived from literature survey (Table 1). In most previous miRNA expression profiling studies of RA and other diseases, miRNA expression was analyzed, and their target genes were predicted and functionally annotated. In the current study, we collected the expression data of RA-related genes from different sources and platforms, and re-analyzed the expression of the miRNA target genes. Our results indicate that studies on genes associated with the molecular mechanism of RA pathogenesis might provide new insights into the underlying cause or regulation of this disease.

Several miRNA target genes are aberrantly expressed in RA [71]. For example, *IKBKE*, which encodes the matrix metalloproteinase production-related protein, is the target gene of miR-155, and their negatively correlated expression has been verified [72]. *TAB2*, *TAB3*, and *IKK-a* are regulated by miR-23b, and their expression has been validated. Differential expression of these target genes inhibits inflammatory cytokine expression and eventually, represses autoimmune inflammation [35]. Upon identification of the potential RA-related miRNAs, their target genes and the corresponding functional signal pathways can be predicted. However, to date, it is technically difficult to verify all genes that show opposite expression variation to miRNAs in RA using a large-scale method. In our analysis, the expression of DEGs in RA was assessed using the collected microarray and RNA-seq data. Interestingly, several negatively-correlated miRNA/mRNA pairs in RA were validated in our study, including miR-19/TLR2, miR-23/TAB3, as well as miR-323 and its target genes GSK3B, BTRC, etc, though the gene expression variation may be less than 2-fold (not shown). In addition, we have identified several potential miRNA target genes (Tables 2–4), and examined the association between the expression of these genes and miRNA regulation. The genetic interaction networks of these negatively correlated mRNA/miRNA pair candidates are shown in Fig 2. The expression profiles for specific genes are discussed below.

*B4GALT1*: Our RNA-seq analysis revealed that *B4GALT1* expression is upregulated in RASFs. This result is consistent with the findings of Wang Y et al., who performed a comparison analysis between RA and OA or HC [73]. *B4GALT1* is regulated by miR-124a, and it may play an important role in inflammatory processes in RA.

*SFRP1*: The secreted frizzled-related proteins (SFRPs) are the pivotal antagonists of the Wnt signaling pathway [74]. *SFRP1* is a tumor suppressor gene, and it is downregulated in many tumors and RA [75, 76]. *SFRP1* is regulated by miR-203 and highly expressed in RASFs. It prevents WNT from binding to other proteins. Thus, *SFRP1* may be an attractive marker for RA diagnosis and therapeutic intervention.

*ROR2*: *ROR2* is the target gene for miR-124. Among the potential targets genes, *ROR2* was identified not only from the RA microarray data comparison, but also from the RNA-seq analysis. *ROR2* encodes transmembrane proteins that belong to the receptor tyrosine kinase-like orphan receptor (ROR) subfamily of cell surface receptors. The ROR proteins play a role in cartilage and bone development, and their dysregulation is associated with several human diseases, including RA [77–79]. *ROR2* was significantly upregulated in RA. Bolzoni et al. demonstrated that high expression levels of *ROR2* are correlated with the pathogenesis of multiple myeloma-induced bone disease [80]. In addition, the ROR2 proteins bind to WNT family proteins [81] and the secreted protein, SFRP1. Together, these results suggest that *ROR2* may be used to monitor the Wnt signaling pathway in RA.

*ABI3BP*: ABI3BP is a member of the ABI family of proteins that is upregulated in chronic osteochondropathy Kashin-Beck disease patients [82]. *ABI3BP* is also a susceptibility gene for thyroid tumors [83]. The GO annotations indicate that ABI3BP may bind to heparin and collagen. Notably, FNDC1 (fibronectin type III domain containing 1) is an important homologue of ABI3BP. Moreover, FNDC1 may be associated with RA pathogenesis [84].

*SMOC2*: The modular extracellular calcium-binding protein SMOC2 is involved in ion binding. It may function as an angiogenic factor that potentiates the angiogenic effects of growth factors [85]. Genome-Wide Association Study (GWAS) has indicated that *SMOC2* is a risky gene locus for generalized vitiligo, which occurs concomitantly with RA and other autoimmune diseases [86]. *SMOC2* is a potential target gene of the dysregulated miR-19b. The expression level of *SMOC2* increased significantly (in GSE21959, fold change > 2, p < 0.05) in RA, compared with HC.

*FAP*: The fibroblast activation protein FAP is a homodimeric integral membrane gelatinase that may be involved in regulating fibroblast growth [87]. *FAP* is targeted by miR-30a. Our results suggest that the expression of *FAP* is increased in RA, compared with HC. A series of studies, including this study, have demonstrated that *FAP* expression is significantly increased in RASFs [88, 89]. *FAP* is also highly expressed in active fibroblastic cells in cancer and hepatic fibrosis [90–92]. *FAP* upregulation eventually affects extracellular matrix degradation, and cartilage and bone destruction in arthritic joints. Therefore, it may be a therapeutic target for the diagnosis and treatment of associated diseases.

RA exhibits both genetic and phenotypic heterogeneity. This generates a clinically heterogeneous condition that impedes investigations on RA pathogenesis and effective diagnostic methods, as well as prognosis and treatment. Recent findings have underscored the role of miRNA as a regulator of RA pathogenesis. Although target genes of dysregulated miRNAs in RA have been predicted and functionally annotated, to our knowledge, detailed analyses of their expression profiles have not been conducted. Moreover, it remains to be elucidated whether the target genes are actually expressed and whether their expression is altered in RA, as well as whether the expression of miRNAs and their target genes are negatively correlated. To the best of our knowledge, this is the first study to identify dysregulated miRNAs in RA and to use bioinformatics approach to obtain RA-related gene expression profiles and test the expression of miRNA-regulated genes. The novel dysregulated target genes identified in RA through integrated analysis of miRNA/mRNA expression may be used as genetic markers for RA diagnosis and treatment in the future.

## Supporting Information

S1 Fig A schema of the current study. (TIF)

S1 Table Functional annotation of differentially expressed genes (DEGs) in series GSE29746. The gene expression profiling of rheumatoid arthritis (RA) was compared with that of healthy control (HC) and osteoarthritis (OA). The potential RA-related DEGs were also checked for their expression between HC and OA, and then underwent a new functional annotation following elimination of DEGs between HC and OA.
(XLS)

S2 Table Functional annotation of differentially expressed genes (DEGs) in series GSE21959. The gene expression profiling of rheumatoid arthritis (RA) was compared with that of healthy control (HC).
(XLS)

S3 Table Functional annotation of differentially expressed genes (DEGs) in series GSE7669. The gene expression profiling of rheumatoid arthritis (RA) was compared with that of osteoarthritis (OA).
(XLS)

S4 Table Functional annotation of differentially expressed genes (DEGs) in SRP009315. The gene expression profiling of rheumatoid arthritis (RA) was compared with that of healthy control (HC).
(XLS)
