# Supplementary figures and images for: Bioinformatics-Based Identification of MicroRNA-Regulated and Rheumatoid Arthritis-Associated Genes
Source: PLoS One. 2015 Sep 11;10(9):e0137551. doi: 10.1371/journal.pone.0137551 (PMC4567271; doi:10.1371/journal.pone.0137551)

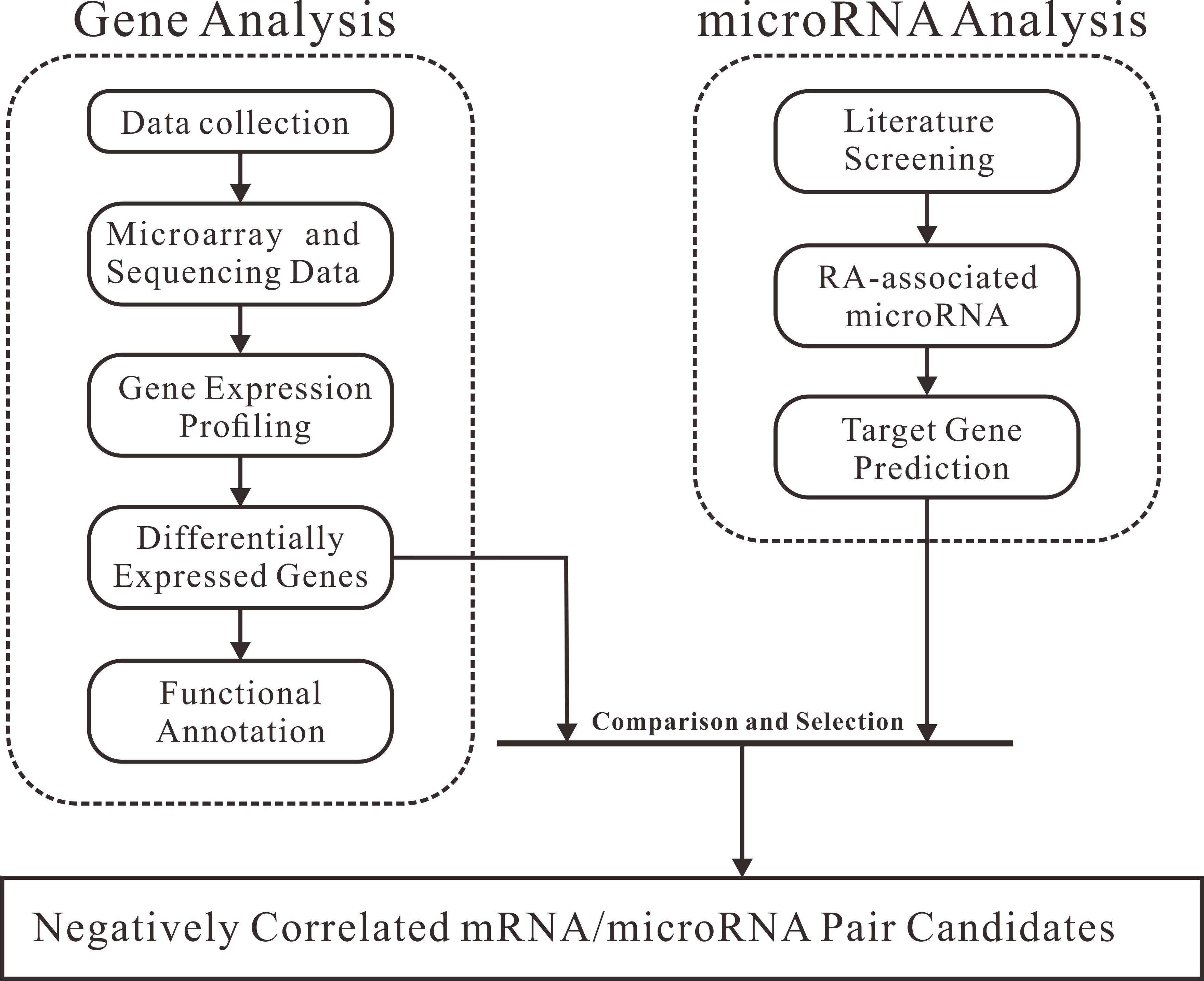

Supplement: S1 Fig — (TIF) [file pone.0137551.s001.tif]
